# The Relationship between Social Capital within Its Different Contexts and Adherence to a Mediterranean Diet Among Lithuanian Adolescents

**DOI:** 10.3390/nu11061332

**Published:** 2019-06-14

**Authors:** Brigita Mieziene, Arunas Emeljanovas, Dario Novak, Ichiro Kawachi

**Affiliations:** 1 Faculty of Sports Education, Lithuanian Sports University, 44221 Kaunas, Lithuania; arunas.emeljanovas@lsu.lt; 2 Faculty of Kinesiology, University of Zagreb, 10000 Zagreb, Croatia; dario.novak@inantro.hr; 3 Institute for Anthropological Research, 10000 Zagreb, Croatia; 4 Department of Social and Behavioral Sciences, Harvard T.H. Chan School of Public Health, Boston, MA 02115, USA; ikawachi@hsph.harvard.edu

**Keywords:** healthy eating, high school students, social support, cross-sectional study

## Abstract

The eating habits of adolescents are a serious current public health problem. Scientists call attention to the availability of social resources for enhancing healthy eating behavior. Social capital defines those resources as trust, reciprocity, social participation, integrity, and coherence, and they are supposed to help people achieve their life goals, in general, and health goals, in particular. Our aim is to investigate the relationship between social capital within its different contexts and adherence to a Mediterranean diet (MD) among Lithuanian adolescents. The nationally representative cross-sectional study included 1863 students (906 boys and 957 girls). The KIDMED index questionnaire (Mediterranean Diet Quality Index in children and adolescents) was used to evaluate the adherence to an MD. Family, neighborhood, and school contexts of social capital were assessed using six items indicating family support, neighborhood trust, social control, vertical trust, horizontal trust, and reciprocity at school. Covariates such as gender, physical activity, parental education, and body mass index were also included in the analysis. Descriptive results showed that only 14% of Lithuanian adolescents followed an MD. Linear regression analysis indicated that family support (β = 0.096) and trust in school teachers (β = 0.074) were related to better rates of adherence, especially regarding the consumption of fruits, vegetables, cereals, fish, and the use of olive oil as a main source of fat. More adolescents who perceived family support and trust in their teachers used these products regularly and were less likely to skip breakfast. These findings could be used as a base for further developing nutrition education programs aimed at enhancing support and trust among families and schoolteachers.

## 1. Introduction

The eating habits of adolescents are a serious current public health problem. Adolescents consume a lot of sweets, baked products, fast food, and sugary drinks [1]. Many do not have breakfast on school days [2]. Such poor eating habits are determinants of cardiovascular disease, diabetes, overweight, and obesity [3], and healthy nutrition is crucial for physical and mental health [4,5,6].

Dietary traditions vary across countries and regions. However, in the scientific literature, a Mediterranean diet (MD) is usually considered healthy and has historically been associated with good health [7]. An MD is not necessarily specific for Mediterranean countries and can be consumed globally, as it focuses not on certain products, but on food groups that are usually available across countries. The key features of an MD are the consumption of plenty of olive oil, legumes, primarily whole-grain bread and cereals, vegetables, fruits, nuts, moderate amounts of dairy products and fish, and low amounts of red meat [8,9].

Studies show that nutrition in adolescence is related to dietary habits in adulthood [10]. A number of authors have recognized that an MD prevents asthma in children [11] and facilitates the control of this disease in both children and adults [12,13]. Theoretically and practically, an MD is a measure of healthy nutrition [14,15].

A series of recent studies has indicated that an MD is a preventive factor for many chronic mental and physical disorders in adults, including depression, Parkinson’s disease, certain types of cancer, myocardial infarction, high blood cholesterol levels, and diabetes [16,17,18]. Furthermore, several studies have suggested that an MD enhances working and long-term memory and prevents dementia and Alzheimer’s disease in the elderly [19,20,21].

Although Lithuania is not a Mediterranean country and is situated in Eastern Europe, it has transitioned to a market economy, 30 years after its independence in 1990. As a result of market globalization, consumption of traditional foods declined, and healthy food options such as vegetable oil, skimmed milk products, fruits, and vegetables became available; however, so did unhealthy foods such as fast food. Changes in the food market impacted the dietary intake of Lithuanians, particularly the quality of fat used [22], and the current generation has already been raised with these new dietary traditions.

It is important to identify the correlates of adherence to an MD in order to understand and provide the basis for behavioral change. A literature review reported that sociodemographic factors such as high socioeconomic status and higher parental education and income levels are associated with higher adherence to an MD, whereas living in an urban environment and being obese are associated with lower adherence [23,24]. Other types of health-related behavior, particularly higher physical activity, are also associated with adherence to an MD [23]. Socioecological models explain that determinants of health behavior depend on different levels, including intrapersonal, interpersonal, institutional, community, and policy levels [25]. Socioecological models provide a framework to better understand the multiple factors and barriers that impact dietary behaviors [26]. Individual factors such as self-efficacy and beliefs play an important role in adherence to a healthy diet. However, knowledge regarding healthy foods and key healthy eating principles do not play a significant role in following healthy eating recommendations [27]. Barriers to healthy eating include a lack of time, limited availability of healthy foods in schools, and a general lack of concern for healthy eating recommendations [27].

The spectrum of sociodemographic and individual factors that affect eating behavior is quite broad. Nevertheless, scientists report that the availability of social resources to enhance healthy behavior is important. They emphasize that behavior change is not effective without considering the social context of the behavior [28] that is represented by the interpersonal, institutional and community levels of the socioecological model. However, there is little evidence that supports the role of social factors in healthy eating. Some studies have concluded that family support is related to higher self-efficacy regarding nutrition behavior among adult churchgoers and Internet users. This behavior, in turn, was associated with higher fiber, fruit, and vegetable consumption and lower fat intake [29,30]. Similar results were obtained among myocardial infarction survivors [31]. However, the majority of prior research has only explored the social correlates of nutrition in adults. Actually, healthy nutrition is crucially important during childhood and adolescence, a period of physical and mental development. This is also a period in life when habits develop, and a close social environment plays a significant role in habit formation.

Social capital outside the family includes school and neighborhood social contexts, partly represented by school and community levels within the socioecological models, altogether brought into the concept that represents social support, trust, reciprocity, social participation, integrity, cohesion, and trust available for the members of the community [32]. Social capital is multi-contextual as well as multi-leveled. Social capital that results from interactions of a group with a person represents ‘bonding’ social capital. Interaction among different groups of the same social scale is considered ‘bridging’ social capital, while interactions among groups of different social scales regarding power are called “linking” social capital [33].

Research suggests that people with higher social capital in its different contexts have higher perceived emotional and physical well-being and healthier behavior patterns [34,35,36,37,38]. The impact of social capital on health behavior may be caused by knowledge about healthy behavior patterns; informal social control, when health-related behavior is encouraged; and psychological processes, when emotional support is provided [39]. However, to the best of our knowledge, there is no scientific evidence that social capital influences adolescents’ eating behavior, and no study has examined the importance of social capital on healthy eating, in general, and with regard to adhering to an MD, in particular. However, several attempts have been made to investigate the relationships among social capital and physical activity [40,41,42] and substance use [43,44,45,46] in adolescents. Although the evidence strongly suggests that high levels of social capital enhance physical activity in adolescents, the results have not been very consistent in regard to social capital and substance use.

Therefore, it is important to study factors that might affect eating habits in adolescents in close social environments such as families, neighborhoods, and schools in order to plan and implement nutrition education programs. The aim of the present study was to investigate the relationship between social capital within its different contexts and adherence to an MD among Lithuanian adolescents.

## 2. Materials and Methods

### 2.1. Study Design

A nationally representative cross-sectional study was performed among Lithuanian adolescents.

### 2.2. Setting

The study sample was gathered from all 10 regions of Lithuania in November and December 2016 and represents both urban and rural areas proportionally. Cluster (area) random sampling was used. Two randomly selected schools (primary sampling units) were selected: one from the main city and one from the rural area of each region. Twenty-six schools were selected in total. Six schools refused to participate in the study. Students from the 9^th^, 10^th^, 11^th^, and 12^th^ grade, or in the first to fourth year of high school (secondary sampling units) were chosen. All students in the selected schools and grades were included in the study. Both the school and classes in the schools were considered clusters. The study procedure took approximately 30 min.

### 2.3. Participants

The study participants were 1928 adolescents with an age range of 14–18 years. Only students who did not wish to participate or whose parents did not give consent were excluded. Among all included students, 15 (0.8%) did not wish to participate, and 50 (2.7%) returned the questionnaires with incomplete data. The rest of the sample, consisting of 1863 students (906 boys and 957 girls), was included in the analysis. All included participants and their parents gave their informed consent for inclusion before they participated in the study. The study was conducted in accordance with the Declaration of Helsinki, and the protocol was approved by the Ethics Committee of Lithuanian Sports University (No. SMTEK-13). Students anonymously completed the paper questionnaires in the classroom. Researchers explained the aim and procedures of the study before the questionnaires were completed. 

### 2.4. Measurements

#### 2.4.1. Dietary Habits

The KIDMED index questionnaire (Mediterranean Diet Quality Index in children and adolescents), which measures Mediterranean dietary patterns in adolescents, was used to evaluate healthy eating. Among 16 items in total, 12 indicate patterns of healthy eating (e.g., consumption of oil, fish, fruits, vegetables, cereals, nuts, pulses, pasta or rice, dairy products and yoghurt), and four reflect unhealthy eating (e.g., consumption of fast food, baked goods, and sweets, and skipping breakfast). Binarized “Yes” and “No” answers are provided. The answer “Yes” for a healthy eating pattern is scored +1, and “Yes” for a negative eating pattern is scored −1. According to the KIDMED index, a score of 0–3 reflects poor adherence to an MD, a score in the range of 4–7 reflects average adherence, and a score of 8–12 reflects good adherence [47,48].

#### 2.4.2. Social Capital

Family, neighborhood, and school contexts of social capital were assessed. Family support was assessed using the question: “Do you feel that your family understands and cares about you?” Neighborhood trust was assessed using the question: “Do you feel people trust each other in your neighborhood?” Informal social control was assessed using the question: “Do you feel that neighbors criticize high school youth engaged in antisocial behavior?” School social capital in terms of vertical school trust was assessed using the question: “Do you feel that teachers and students trust each other in your high school?”, in terms of horizontal trust using the question: “Do you feel students trust each other in your high school?”, and in terms of reciprocity using the question: “Do you think students collaborate with each other in your high school?”. The responses were ranked on a five-point Likert scale as follows: (1) strongly agree, (2) agree, (3) neither agree nor disagree, (4) disagree, and (5) strongly disagree. The answers were binarized into “high” (strongly agree and agree) and “low” (neither agree nor disagree, disagree, and strongly disagree) social capital [49]. These items have been previously used in the studies examining relationships of social capital and physical activity among adolescents of Turkey [40] and Croatia [42], examining relationships of social capital and self-rated health among adolescents in Serbia [50] and Lithuania [51] and in combined sample of European adolescents [52]. 

### 2.5. Covariates

Physical activity was assessed using the short version of the International Physical Activity Questionnaire. The indicator was metabolic equivalents (METs) of hours per week [53]. Height and weight were self-reported, and body mass index (BMI) was calculated. Participants were divided into not overweight and overweight or obese groups according to age and gender-adjusted overweight and obesity thresholds [54]. Parental education was indicated by the parents choosing among the answers “Elementary school”, “Middle school”, “High school”, “Vocational training”, and “College/University”. Gender was also considered as a covariate.

### 2.6. Statistical Analysis

Data were analyzed using SPSS 24.0 software (SPSS Inc., Chicago, IL, USA). Descriptive statistics were employed to determine means and percentage distribution of variables used in the study. The chi-square test was used to identify relationships between nominal and categorical study variables. Fisher exact probability test for a two-rows by three-columns contingency table was applied in appropriate cases. The relationships between social capital contexts and dietary patterns were identified using hierarchical linear regression analysis, controlling for the covariates gender, BMI, parental education, and physical activity. Statistical significance was set at a *p*-value of less than 0.05.

STROBE Statement-checklist guidelines were followed in organizing this paper.

## 3. Results

Descriptive statistics of the study sample are summarized in Table 1 and indicate that male students had asignificantly higher mean of adherence to an MD than did female students (*p* < 0.01). The proportion of poor adherence to healthy eating patterns was significantly higher in females than in males (*p* < 0.05), and the proportion of good adherence was higher in males than in females (*p* < 0.05). Almost equal proportions of male and female students were in the group of average adherence, i.e., almost half of the students of each gender. No significant difference was found between gender groups comparing average adherence to an MD (*p* > 0.05). This tendency replicated itself for physical activity. Male students were more physically active than were female students, accumulating 1138.48 more METs per week (*p* < 0.01). Male students also had a significantly higher BMI than did female students (21.8 vs. 20.60, respectively) (*p* < 0.01) and 4.6% more of the male than female students were overweight or obese (*p* < 0.01). The perception of family support and reciprocity at school were similar in male and female students (*p* > 0.05); however, more males than females perceived high neighborhood (11.2% difference) (*p* < 0.01), vertical (6.8% difference) (*p* < 0.01), and horizontal (12.4% difference) (*p* < 0.01) trust. Meanwhile, significantly more female than male students perceived high social control (5.2% difference) (*p* < 0.05).

Hierarchical linear regression was employed to identify the predictive values of social capital indicators for healthy diet, controlling for sociodemographic factors, physical activity, and BMI as variables. The results in Table 2 indicate that a higher perception of family support was significantly related to better dietary patterns in adolescents (β = 0.096, *p* < 0.01); the same could be said about vertical trust (β = 0.074, *p* < 0.01). The more students perceive that they can trust or rely on their teachers in school, the better dietary habits they develop. Neither neighborhood trust, social control, nor peer-related horizontal trust and reciprocity at school were associated with dietary habits (*p* > 0.05).

Among the controlling factors (Table 2), female gender was associated with lower adherence to healthy eating patterns through all three models significantly (*p* < 0.01). Parental level of education was significant through all steps only for the fathers (*p* < 0.01). In the third model, when physical activity, BMI, and social capital were added as variables, the standardized β was reduced from 0.154 to 0.139. The higher the father’s education, the higher the adherence of the child to a healthy diet. The mother’s education was significant in only the first model (β = 0.065, *p* < 0.05), but not in the second model after BMI and physical activity were added (*p* > 0.05). BMI was not a significant predictor of adherence to an MD (*p* > 0.05). Higher physical activity was related to better adherence to healthy eating patterns, independent of social capital variables (β = 0.188, *p* < 0.01). Meanwhile, older age was related to lower adherence to healthy eating (β = −0.053, *p* < 0.05), unless social capital variables were added in the third model (*p* > 0.05).

A comparison of specific dietary habits (Table 3) in low and high social capital groups in general confirmed previous results that a higher perception of social capital is related to better dietary habits. More high school students with a high perception of family support, neighborhood trust, vertical and horizontal trust, and reciprocity at school regularly ate fruits (the differences varied from 7.3% to 14.3% between low and high social capital across domains, *p* < 0.01) and vegetables (the differences varied from 6.8% to 7.6% between low and high social capital across contexts, *p* < 0.05) compared with students that reported lower perception. Cereals and grains were consumed more often by those who perceived high family support (with a difference of 9.5%, *p* < 0.01) and vertical trust (with a difference of 4.2%, *p* < 0.05) than among those with low support. Among those with high family support (with a difference of 17.2%, *p* < 0.01), neighborhood trust (with a difference of 4.4%, *p* < 0.05), horizontal trust (with a difference of 4.6%, *p* < 0.05), and reciprocity at school (with a difference of 7.8%, *p* < 0.05), olive oil was a main source of fat compared with students with low social capital across those contexts. The use of dairy products was also increased among those with high family support (with a difference of 9.2%, *p* < 0.01), social control (with a difference of 5.5%, *p* < 0.01), and perception of reciprocity at school (with a difference of 4.9%, *p* < 0.05) compared with students with low social capital across those contexts. In addition, students who perceived low family support ate more fast food (with a difference of 6.2%, *p* < 0.05), skipped breakfast more frequently (with a difference of 5.3%, *p* < 0.05), and ate commercially-baked goods or pastries for breakfast (with a difference of 6.1%, *p* < 0.05) more often than students with high family support. Skipping breakfast was also more common among students who perceived low compared with high vertical trust (with a difference of 5%, *p* < 0.01), reciprocity at school (with a difference of 5.8%, *p* < 0.05), and high vs low social control (with a difference of 6.5%, *p* < 0.01). Consumption of commercially baked goods or pastries for breakfast was also more common in students with a low perception of reciprocity at school compared with those with a high perception (with a difference of 5.5%, *p* < 0.01). Daily consumption of sweets was related only to the perception of high social control (with difference of 5.7%, *p* < 0.05) among students with low vs high social control.

## 4. Discussion

This study aimed to examine the relationships between social capital in family, neighborhood, and school contexts and adherence to an MD among high school students from Lithuania—a non-Mediterranean country. To the best of our knowledge, this is the first study to examine the relationship between social capital and eating behavior in adolescents.

The results showed that only 14% of Lithuanian high school students have good adherence to an MD, nearly half have average adherence, and around 40% have poor adherence. Research has shown that adherence to an MD is low, even in Mediterranean countries. Low rates of adherence among adolescents have been reported in Greece [55]. Only 8.3% of Greek adolescents aged 13–18 years had good adherence, while 68.6% had average adherence, and 27% had poor adherence to an MD [56]. A review revealed that roughly half of the adolescents in Mediterranean countries have average adherence to an MD, while nearly half may have a trend toward poorer adherence [24]. Remarkably lower rates of adherence have been reported in the Tuscan region, where only 16.5% of adolescents have good adherence, 60.5% have average adherence, and 23% have poor adherence to an MD [57]; this seems to be a common problem in non-Mediterranean countries as well. For instance, in Iceland, only 24.3% of adolescents have good adherence to an MD [58]. Adherence is even worse in a common combined sample of Lithuanian and Serbian adolescents. Only 13.3% of these adolescents have good adherence to healthy eating patterns, and boys show better adherence than do girls [59]. In the United Kingdom, the results of a large population-based study of 9–10-year-old children indicated that only 6% achieved the highest points on the MD questionnaire [60]. The results of another study have shown better trends in another non-Mediterranean country, Portugal. Almost half (48.74%) of Portuguese adolescents had high adherence to an MD [61]. These results are similar to their Mediterranean neighbors, Spanish adolescents. More than 40% of the youth in Spain [47,62] and half of the adolescents in the Balearic Islands [63] had good adherence to an MD, almost half had average adherence, and just a small portion (2–4%) had poor adherence [47,62]. These diverse results across both Mediterranean and non-Mediterranean countries suggest again that an MD is no longer associated with living in the Mediterranean area. The Mediterranean countries are also affected by food market globalization. Especially their young generations already adopted patterns of Western diet and in general trends in dietary habits in these countries change towards unhealthy pattern [24]. Moreover, other health-related behaviors such as physical activity and time spent in sedentary behaviors are also raising concerns in many countries, including those listed above [64]. These trends seem to be associated with the process of globalization and socioeconomic inequalities rather than geographical area [65].

Almost nine out of 10 adolescents perceived high family support. These results indicate that adherence to an MD among Lithuanian adolescents is better when they perceive more family support. In particular, adherence to an MD is associated with the consumption of fruits, vegetables, fish, dairy products at breakfast, and the use of olive oil at home. Furthermore, these adolescents eat commercially-baked goods or pastries for breakfast and go to fast food restaurants less frequently. A systematic literature review also revealed that healthy eating patterns are related to family support [66]. Other recent research has reported that a parental example of fruit and vegetable consumption is a significant predictor for similar behaviors in adolescents [67]. Empirical evidence has provided deeper details suggesting that descriptive norms (what parents do) are more important than injunctive norms (what parents say) in the case of eating fruits and vegetables among Danish adolescents (mean age of 12.5 years) [68]. Adherence to an MD in the present study was also higher among Lithuanian adolescents whose fathers had a higher education level. Grosso and Galvano [23] explained that family environments with a better social background may be more supportive and caring and might encourage healthier eating habits and behaviors. Research indicates that fruit and vegetable consumption is primarily related to their availability at home, which is particularly important for children with low preferences for healthy food choices [69].

In the present study, half of the students perceived high trust in their teachers. Adjusting for sociodemographic variables, BMI and physical activity indicated that these adolescents had better dietary patterns than those with low trust. Specifically, high trust in teachers was related to more frequent consumption of fruits, vegetables and fish, and a lower frequency of skipping breakfast. Other studies have investigated the relationship between student trust in their teachers and other types of health behaviors. One study found that a lower vertical trust among Croatian high school boys was associated with regular overall physical activity [42]. Kawachi and Berkman [39] reported that the link between social capital and health behavior might develop in several ways. First, on the basis of knowledge provided about health-promoting behavior, compulsory health education at school provides the main information on health-enhancing physical activity and healthy eating. Second, on the basis of nonformal social control, when health behavior is promoted creating a health behavior friendly environment. For example, provision of healthy food in the school canteen and no options of unhealthy food choices available nearby. Third, on the basis of psychological processes, providing emotional support, relatedness, and respect is important, as it is a key issue for developing trust between teachers and school students. It can also be speculated that personality traits, such as conscientiousness, are moderators that may explain why more conscientious students trust their teachers more and tend to eat healthier [70]. Therefore, personality traits as moderators important for the relationship between social capital and eating behaviors should be explored in additional empirical studies.

Regression analysis also revealed that physically active students were more likely to eat healthier than physically inactive students. This result may reflect conscious behavior in students, i.e., conscientious students may tend to engage more in health-related behaviors in general [70]. In line with these results, it has been shown that adherence to an MD in children and adolescents in southern European countries is associated with higher physical activity and less time spent in sedentary behaviors [23,24].

No relationship was seen between peer-related factors and adolescents’ adherence to an MD. Neither horizontal trust nor reciprocity at school had a significant effect on the MD score. A systematic review on barriers and facilitators of young peoples’ healthy eating revealed that social spaces and friends are not always a source of information and support for healthy eating [66]. However, more detailed analyses in the present study revealed that those students who had higher horizontal trust and reciprocity at school consumed fruits and vegetables more frequently. These results are in line with a recent study involving 8^th^- and 11^th^-grade students in Texas (USA). Researchers found that peer support was related to higher odds of daily consumption of fruits and vegetables [67]. Although peer support in the Texas study was measured by indicating the descriptive norm, i.e., if peers were eating fruits and vegetables themselves, the present study measured whether peers could be trusted in general and if there was collaboration among peers at school. Still, the social component of eating might link these different measurements. Adolescents spend a lot of time doing things together at school, and they probably eat together with those whom they trust and collaborate. The patterns of eating at school probably become similar among close friends.

In the present study, Lithuanian girls demonstrated lower adherence to an MD than did boys. Other studies have presented mixed evidence regarding the role of gender in eating patterns. No gender difference in adherence to an MD was found in other non-Mediterranean countries, including Iceland (13–16-year-olds) [58], Sicily (13–16-year-olds) [23], Greece (10–12-year-olds) [55], and Spain (2–24-year-olds) [47]. Among Greek school-age children, girls 3–18-years-old showed slightly higher adherence to an MD than did boys [56]. In Italy, adherence seems to be a bigger problem among females than among males, as more males (58.5%) compared with females (41.5%) have high adherence to an MD [23]. Male gender has also been reported to be a significant predictor of higher adherence to an MD in 2–5-year-old boys [71]. Reviews of studies on an MD have reported that boys and girls who are more physically active consume breakfast cereals, fresh fruits, and yogurt more frequently [24]. Given that in the present study, boys were more physically active than were girls, it may be that they also consumed more food that is considered healthy.

Many studies have indicated the importance of parental education in the healthy eating choices of their children [24], and the present study also found that higher paternal education was related to higher adherence to an MD among Lithuanian adolescents. The mothers’ education was positively related to adherence to an MD only until BMI was added into the regression analysis. Studies have shown that adherence to an MD is not always associated with a better weight status [55]. In the present study, BMI was not a predictor of adherence to an MD. A review of other studies revealed that overweight and obese youngsters were less likely to adhere to an MD [24].

### Strengths and Limitations

The present study did have some limitations. First, the cross-sectional nature of the study may be considered a weakness, as no causal relationships can be drawn from the results. Second, both health-related behaviors—adherence to an MD as well as physical activity—were measured subjectively. This could have led to an overestimation of these behavior patterns [72]. However, our findings are valuable and could be used as the basis for further, deeper analysis. Research on adherence to an MD in non-Mediterranean countries is scarce [73], and our study is important because it might provide evidence explaining the differences in health outcomes across Mediterranean and non-Mediterranean countries. To our knowledge, this is the first study to investigate the impact of social capital on adherence to an MD. The findings could be used as a basis for developing nutrition education programs that aim to improve support and trust from families, peers at school, and schoolteachers.

## 5. Conclusions

Only 14.1% of Lithuanian adolescents analyzed in the present study showed good adherence to an MD, whereas almost half had average adherence and nearly 40% had poor adherence. Among covariates, male gender, higher father’s education, and higher physical activity were related to better adherence to an MD.

Social capital in the family and school contexts are important for better eating patterns. Family support and trust in schoolteachers are related to better rates of adherence to an MD, especially in regard to the consumption of fruits, vegetables, cereals, grains, fish, and the use of olive oil as a main source of fat. More adolescents who perceived family support and trust in their schoolteachers used these products regularly, and fewer skipped breakfast. Trust in peers and reciprocity at school were not important for adherence to an MD in general, but were still related to fruit and vegetable consumption and the use of olive oil as a main source of fat. Neighborhood trust was also related to some positive eating habits, such as consumption of fruits and vegetables, pasta or rice, nuts, and the use of olive oil. Social control in the neighborhood showed mixed relationships with healthy and unhealthy eating patterns.

## Figures and Tables

**Table 1:** Descriptive statistics and comparison of study variables between genders (n = 1863).

Study Variable	Total n (%) or Mean ± SD	Male n (%) or Mean ± SD	Female n(%) or Mean ± SD
*Mediterranean diet*			
Total MD score	4.32 ± 2.77	4.65 ± 2.77 ##	4.01 ± 2.74
Poor	738 (39.6)	316 (34.9)	422 (44.1) **
Average	863 (46.3)	431 (47.6)	432 (45.1)
Good	262 (14.1)	159 (17.5) **	103 (10.8)
Physical activity (MET)	4940.06 ± 3953.90	5529.30 ±4216.46 ##	4390.82 ± 3608.93
*BMI*	21.20 ± 3.14	21.8 ± 3.17 ##	20.60 ± 2.99
Not overweight	1683 (91)	796 (88.5)	887 (93.3) **
Overweight	134 (7.2)	81 (9.0) **	53 (5.6)
Obese	33 (1.8)	22 (2.4) **	11 (1.2)
*Social capital*			
Family support			
Low	226 (12.1)	110 (12.1)	116 (12.1)
High	1637 (87.9)	796 (87.9)	841 (87.9)
Neighborhood trust			
Low	944 (50.7)	407 (44.9)	537 (56.1)
High	919 (49.3)	499 (55.1) **	420 (43.9)
Social control			
Low	1291 (69.3)	652 (72.0)	639 (66.8)
High	572 (30.7)	254 (28.0)	318 (33.2) *
Vertical trust			
Low	926 (49.7)	419 (46.2)	507 (53.0)
High	937 (50.3)	487 (53.8) **	450 (47.0)
Horizontal trust			
Low	859 (46.1)	360 (39.7)	499 (52.1)
High	1004 (53.9)	546 (60.3) **	458 (47.9)
Reciprocity at school			
Low	431 (23.1)	207 (22.8)	224 (23.4)
High	1432 (76.9)	699 (77.2)	733 (76.6)

Note: *—*p* of Chi-square test is <0.05; **—<0.01; ##—*p* of Student’s *t* test is <0.01.

**Table 2:** Predictors of better adherence to a Mediterranean diet among Lithuanian high school students (hierarchical linear regression).

Variable	Standardized Beta		
	Model 1	Model 2	Model 3
Age	−0.111 *	−0.053 *	−0.041
Gender (female)	−0.048 **	−0.074 **	−0.063 **
School	−0.036	−0.032	−0.025
Father’s education	0.154 **	0.148 **	0.139 **
Mother’s education	0.065 *	0.040	0.027
BMI (overweight and obese)		0.037	0.042
Physical activity		0.194 **	0.188 **
Family support			0.096 **
Neighborhood trust			0.031
Social control			−0.027
Vertical trust			0.074 **
Horizontal trust			0.005
Reciprocity at school			0.005
ΔR	0.057 **	0.038 **	0.022 **

Note: *—*p* < 0.05; **—*p* < 0.01.

**Table 3:** Comparison of percentage distribution of nutrition habits among Lithuanian high school students with low and high social capital (chi-square test).

Nutrition Habit for Response YES	Family Support	Neighborhood Trust	SOCIAL Control	Vertical Trust	Horizontal Trust	Reciprocity at School
	Low/High (%); χ^2^	Low/High; χ^2^	Low/High; χ^2^	Low/High; χ^2^	Low/High; χ^2^	Low/High; χ^2^
Eats a fruit or fruit juice every day	53.1/67.4; 18.12 **	62.1/69.4; 11.15 **	65.7/65.7; 0	61.9/69.5; 11.93 **	60.0/70.6; 23.36 **	57.5/68.2; 16.57 **
Eats a second fruit every day	44.7/54.9; 8.25 **	49.0/58.3; 16.11 **	53.5/53.8; 0.02	49.0/58.2; 15.63 **	49.2/57.4; 12.30 **	49.0/55.0; 4.91 *
Eats fresh or cooked vegetables regularly once a day	46.5/54.1; 4.68 *	49.8/56.7; 8.92 **	52.5/54.7; 0.77	49.8/56.6; 8.60 **	49.0/56.8; 11.20 **	49.0/54.5; 4.05 *
Eats fresh or cooked vegetables more than once a day	27.9/30.8; 0.82	26.8/34.3; 12.28 **	28.2/35.7; 10.43 *	27.8/33.2; 6.50 *	26.7/33.8; 11.03 *	28.8/31.0; 0.78
Consumes fish regularly (at least 2–3 times per week)	16.8/23.2; 4.67 *	19.9/25.0; 6.99 **	22.5/22.2; 0.03	20.2/24.7; 5.32 *	20.3/24.3; 4.36 *	23.2/22.2; 0.19
Goes more than once a week to a fast-food restaurant	24.3/18.1; 4.97 *	19.9/17.8; 1.30	16.7/23.8; 12.84 **	18.7/19.1; 0.54	18.4/19.3; 0.26	20.6/18.4; 1.13
Likes pulses and eats them more than once a week	25.7/29.1; 1.17	26.9/30.6; 3.06 *	27.3/32.0; 4.32 *	27.1/30.3; 2.34	27.1/30.1; 1.98	27.8/29.0; 0.21
Consumes pasta or rice almost every day	45.1/44.2; 0.08	41.5/47.1; 5.90 **	43.5/46.0; 0.96	42.7/45.9; 1.98	43.7/44.8; 0.26	46.6/43.6; 1.26
Eats cereals or grains (bread, etc.) for breakfast	58.4/67.9; 8.00 **	66.0/67.5; 0.52	66.0/68.4; 0.99	64.6/68.8; 3.80 *	66.1/67.2; 0.26	63.6/67.7; 2.50
Consumes nuts regularly (at least 2–3 times per week)	25.7/30.9; 2.59	26.9/33.7; 10.28 **	30.0/30.9; 0.18	26.7/33.8; 11.30 **	27.8/32.4; 4.54 *	29.9/30.4; 0.03
Uses olive oil at home	48.2/65.4; 25.09 **	61.1/65.5; 3.85 *	62.4/65.2; 1.32	62.0/64.6; 1.34	60.8/65.4; 4.35 *	57.3/65.1; 8.62 *
Skips breakfast	28.3/23.0; 3.15 *	25.2/22.0; 2.70	21.6/28.1; 9.39 **	26.1/21.1; 6.46 **	24.3/23.0; 0.45	28.1/22.3; 6.17 *
Eats a dairy product for breakfast (yoghurt, milk, etc.)	46.0/55.2; 6.69 **	53.5/54.6; 0.24	52.4/57.9; 4.84 **	53.0/55.1; 0.79	53.8/54.3; 0.05	50.3/55.2; 3.09 *
Has commercially baked goods for breakfast	23.9/17.8; 4.93 *	18.6/18.4; 0.02	17.1/21.7; 5.46 *	19.1/17.9; 0.43	20.0/17.2; 2.39	22.7/17.2; 6.62 **
Eats two yoghurts and/or some cheese (40 g) daily	30.5/38.3; 5.12 *	35.5/39.3; 2.87	36.8/38.6; 0.58	36.2/38.5; 1.10	35.6/38.8; 2.05	36.2/37.7; 0.33
Eats sweets and candy several times every day	58.4/56.5; 0.29	56.3/57.2; 0.18	55.0/60.7; 5.19 *	56.8/56.7; 0.01	57.3/56.3; 0.19	57.8/56.4; 0.25

Note: significance of χ^2^; *—*p* < 0.05; **—*p* < 0.01.
